# Acute Psychosis and Autoimmune Encephalitis: A Diagnostic Challenge in Clinical Practice

**DOI:** 10.1192/j.eurpsy.2026.11807

**Published:** 2026-07-31

**Authors:** A. Gomez Prieto, A. García Gonzalez, I. Zorrilla Martinez, A. M. González-Pinto Arrillaga

**Affiliations:** Department of Psychiatry, Osakidetza, Vitoria, Spain

## Abstract

**Introduction:**

Anti-NMDA receptor encephalitis is an autoimmune disorder with subacute neuropsychiatric onset. Frequently, it presents with psychiatric symptoms such as delusions, hallucinations, behavioral disturbances, or catatonia, leading to psychiatric admission and delayed diagnosis. Underdiagnosis is associated with worse outcomes, while early immunotherapy improves prognosis.

**Objectives:**

To report a case of anti-NMDA receptor encephalitis initially misdiagnosed as acute psychosis, emphasizing the clinical and paraclinical features guiding the autoimmune suspicion and key differential diagnoses.

**Methods:**

Clinical course, treatments, investigations, and multidisciplinary approach of a 42-year-old woman involuntarily admitted to an acute psychiatric unit were reviewed. She presented with two weeks of disorganized behavior, incoherent speech, bizarre delusions, distressing auditory hallucinations, and inappropriate laughter. Family history revealed acute stress prior to onset. During admission, fluctuating disorientation, cognitive and memory impairment, agitation, and variable consciousness were observed.

**Results:**

The patient showed no improvement after three weeks of antipsychotic treatment. Red flags for organic etiology included fluctuating course, early cognitive deficits, isolated fever without infection, and lack of psychotropic response. Differential diagnoses included affective psychosis, late-onset schizophrenia, metabolic encephalopathy, CNS infection, and other autoimmune encephalitides. Investigations revealed normal labs except iron-deficiency anemia, negative toxicology, normal CT/MRI, nonspecific EEG abnormalities, and CSF pleocytosis with positive anti-NMDA antibodies in serum and CSF. Oncological screening was negative. Immunotherapy with methylprednisolone and IV immunoglobulins was initiated, achieving partial stabilization.

**Conclusions:**

Autoimmune encephalitis should be suspected in atypical psychiatric presentations with subacute onset and antipsychotic resistance, even when neuroimaging is normal. Key warning signs are fluctuating consciousness, disorientation, early cognitive decline, disproportionate memory deficits, atypical psychotic features, and poor response to medication. Early recognition and close collaboration between psychiatry, neurology, and internal medicine are crucial to avoid diagnostic delays and improve prognosis.

1. Dalmau J, et al. Anti-NMDA-receptor encephalitis: case series and analysis of the effects of antibodies. Lancet Neurol 2008;7:1091–8.
2. Graus F, et al. A clinical approach to diagnosis of autoimmune encephalitis. Lancet Neurol 2016;15:391–404
3. Al-Diwani A, et al. The psychopathology of NMDAR-antibody encephalitis in adults: a systematic review and phenotypic analysis of individual patient data. Lancet Psychiatry 2019;6:235–46.

**Disclosure of Interest:**

None Declared

